# Quantify unmet medical need across the disease landscape – A large language model-based methodology

**DOI:** 10.1371/journal.pmed.1004798

**Published:** 2026-03-12

**Authors:** Elliott W. Sharp, Nicholas Fragola, Charlotte Blewitt, Matthew Goddeeris, Lee Lancashire, Charlie Hempstead, David C. Fajgenbaum

**Affiliations:** Every Cure, Philadelphia, Pennsylvania, United States of America; University of California Los Angeles, UNITED STATES OF AMERICA

## Abstract

**Background:**

Despite the ultimate goal of medical researchers and funders being to maximize patient benefit, there is no systematic process for quantifying unmet medical need across diseases. While a relative unmet medical need scoring system would be valuable for prioritization of medical research, systematically performing this effort across all 22,701 human diseases is technically challenging, time-consuming, and expensive. Using a large language model-based (LLM) architecture, we built a scalable method demonstrating feasibility to quantify “unmet medical need” criteria across all diseases, combine those criteria into a single weighted score, and extend the method into new criteria or diseases in the future. We aimed to quantitatively determine which diseases have the greatest unmet medical need and, therefore, which diseases are priority targets for new repurposed treatments.

**Method and findings:**

We defined 11 scoring criteria across three categories of unmet medical need. For each criterion, we tested LLM models and refined prompts to generate a score per criteria for each disease and then defined a weighting for each criterion to contribute to a final score. A 30-disease development set was used to iterate on the prompting, and a 10-disease evaluation set was held out and used to evaluate the performance of the final prompt. All 22,701 human diseases in the MONDO disease ontology were quantitatively scored for their unmet medical need across all 11 weighted criteria. The resulting scores allowed for relative comparison between diseases of unmet medical needs. Inter-expert agreement was strong, indicating reliability of the scoring framework with 95% of ratings within a 1-point difference. Across multiple LLMs, gpt-4o is most closely aligned with expert rankings, achieving low mean and standard deviation differences relative to human scores. Furthermore, LLM-generated scores demonstrated strong Spearman’s rho correlations with expert assessments across key clinical criteria, such as mortality (ρ = 0.845) and quality-adjusted life years lost (ρ = 0.822), supporting their suitability for prioritizing unmet medical need. All data were generated in ~1 hour with no missing data, at a total cost of $120 USD of compute and the results of the Unmet Medical Need Index are publicly available. The main limitation of this study is the combined size of the development and evaluation set being 40 diseases.

**Conclusions:**

This accessible, scalable methodology enables funders and researchers, across governments, universities, healthcare organizations, and disease groups to tailor prioritization efforts according to unmet medical need in the context of their organizational objectives, by selecting appropriate criteria and weighting of those criteria. This method creates a pragmatic and transparent tool to streamline research prioritization. Future research should consider expanding the disease set size used to create scores.

## Author summary

**Why was this study done?:**

- We identified that there is currently no global, systematic database or technique that measures and ranks the “unmet medical need” across all known human diseases.
- Relative scores of unmet medical need would help with prioritizing research activities across diseases, but manually assessing all diseases is impractical because it is too expensive and requires years of specialized expertise.
- A scalable method is needed to help organizations prioritize their funding and research toward the diseases where new treatments will have the greatest impact on saving and improving patient lives.

**What did the researchers do and find?:**

- We developed an artificial intelligence method that evaluates 11 different factors across three areas (patient suffering, standard of care, and accessibility) to create an “unmet medical need score” for every known disease.
- We found that this system could accurately score 22,701 diseases in ~1 hour for a total computing cost of only $120.
- Our analysis showed that the scores generated by artificial intelligence closely matched the rankings provided by a panel of medical experts.

**What do these findings mean?:**

- We have provided an accessible and low-cost method that allows governments, universities, and nonprofits/charities to more objectively understand the relative unmet medical need across diseases, which could be used to prioritize diseases in need of research attention.
- This method is highly flexible, allowing different organizations to adjust the importance of specific factors, such as prioritizing rare childhood diseases or those with the highest death rates, to match their specific mission.
- While this system is a powerful guide for comparing thousands of conditions, we recommend using the scores in combination with other methods rather than as a final, standalone judgment.
- The main limitation of this study is the combined size of the development and evaluation set being 40 diseases, which is helpful to be able to develop scores quickly, but means the ability to generalize these results is limited.

## Abstract 3

Elliott W Sharp and colleagues develop Large Language Model-Based Methodology to quantify unmet needs among human diseases considering factors related to patient suffering, standard of care, and accessibility of care.

## Introduction

Funders of medical research and researchers themselves often aim to maximize the patient benefit from their investments of time and funding. To achieve this aim, research is typically focused on diseases that are perceived to have high unmet medical need. Every Cure is a nonprofit organization using artificial intelligence (AI) to search for high-potential drug repurposing opportunities that address conditions with the greatest unmet medical need and would thus benefit from being able to quantify unmet medical need to assist with prioritizing among millions of promising disease treatments [1,2].

However, there is no systematic approach or agreed upon database that scores the unmet medical need across all diseases. Creating this is challenging, expensive, and time-consuming for three main reasons. There is a significant technical challenge involved in mapping between datasets with different disease names [3]. Furthermore, ongoing maintenance of this data is required because variables change over time such as the standard of care for a disease. Finally, particularly in the rare disease space, existing databases often miss some diseases and may lack comprehensive data or exhaustive clinical descriptions; large language models (LLMs) can help bridge these gaps by synthesizing information across disparate sources [4].

Modern LLMs are well suited to address these problems, though their limitations such as hallucinations and lack of reliable source attribution must be managed. For example, rare disease names are sometimes descriptive of the clinical findings associated with the disease (e.g., anophthalmia-megalocornea-cardiopathy-skeletal anomalies syndrome) and if an LLM indexes on one of the descriptive terms, rather than considering the disease name as a whole, it might generate incorrect outputs because of the probabilistic rather than deterministic nature of LLMs. Nevertheless, there are several advantages to using LLMs with appropriate prompt-engineered architecture to quantify unmet medical need for diseases. A well-designed architecture provides transparency and control by allowing users to see and refine the specific criteria and weightings used to calculate disease scores. Furthermore, the huge datasets LLMs have been trained on include multiple disease definitions, enabling them to recognize and return information using any number of disease identifiers, instead of being dependent on manual mapping of one disease identifier to any other [5]. In addition, LLM-based approaches can be re-run with limited additional time or cost, enabling easy maintenance of scores as medical understanding of diseases changes over time. It is similarly straightforward to add new diseases or new criteria. Finally, where a single database may not contain data on a disease or a disease characteristic, LLMs have access to their full training data for any given disease and will provide a score for every criterion and disease, even where that information is less certain, though caution should be exercised in these cases.

Herein, we aimed to quantitatively determine which diseases have the greatest unmet medical need and, therefore, which diseases are priority targets for new repurposed treatments. Although the method here is used in the context of unmet medical need, this applied AI method is translatable to create many different types of AI-based datasets.

## Methods

The primary aim was to quantitatively determine which diseases have the greatest unmet medical need and therefore which diseases are priority targets for new repurposed treatments.

To achieve this aim, we developed a reproducible, scalable methodology utilizing the corpus of knowledge contained within LLM training data, organized into 10 reproducible and sequential steps.

### Step 1: Capturing all disease names

To ensure all disease names are captured, and precisely define what is a “disease”, we used the MONDO disease ontology, which is “a semi-automatically constructed ontology that merges in multiple disease resources to yield a coherent merged ontology”, encompassing 22,701 human diseases at the time of writing [6]. The primary benefit of MONDO in this context is that the ontology is inclusive of many less frequently diagnosed diseases.

### Step 2: Defining key contributory factors of unmet medical need

We identified three overarching categories influencing unmet medical need for drug repurposing through our work to date and by considering relevant disease burden frameworks such as Global Burden of Disease and the EU Orphan Disease guidelines: patient suffering, current standard of care, and accessibility of the standard of care [7,8]. Each category was further subdivided into three to four specific questions, resulting in 11 quantifiable factors:

- **Patient suffering**: disease prevalence/incidence; duration of illness; quality adjusted life years (QALYs) lost (i.e., morbidity); and 5-year mortality rate.
- **Standard of care**: disease modifying activity; adverse events/side effects; administration route; and administration frequency.
- **Accessibility**: cost to patients; supply robustness; and regulatory barriers.

### Step 3: Creating representative development and evaluation sets

Next, we chose to select 40 diseases for LLM calibration and performance evaluation. The selection of diseases for this analysis was conducted by a multidisciplinary panel of three experts (ES, NF, and CB). The panel comprised a physician (ES) with broad training across surgical and medical specialties and experience in life sciences strategy consulting; a pharmaceutical scientist (NF) specializing in neuropsychiatric disorders and healthcare strategy consulting; and a specialist in biomedical informatics (CB) specializing in neurodevelopmental disorders with a focus on data analysis.

ES, NF, and CB selected 40 diseases to represent a spectrum of unmet medical need based on their experiences from patients in clinical and research settings, with 30 (75%) being used for LLM calibration (development set) and 10 (25%) being used to evaluate LLM performance (evaluation set). A small number of diseases were intentionally chosen to demonstrate that prompts and datasets can be developed in resource-constrained settings and rapid development is possible for this proof of concept, with a larger and more diverse sample being necessary to further validate the proof of concept in the future.

These diseases represented the full spectrum of expected scores for each criterion, as determined by the three medical experts. These diseases were selected through four methods and a subsequent reconciliation of diseases was conducted to ensure an appropriate mix of disease characteristics.

The first method focused on diseases that are common (high-prevalence or incidence) but typically cause less suffering per affected person, as measured by Global Burden of Disease metrics. This method helped identify diseases included such as stroke, breast cancer, diabetes, dermatitis, asthma, chronic obstructive pulmonary disease (COPD), and prostate cancer [7].

The second method was to identify diseases which are rarer but typically cause more suffering per affected person by considering the EU Orphan Disease registry which contains more rare diseases. This method helped identify diseases such as sickle cell disease, glioblastoma, and pancreatic cancer [7,8].

Given that this scoring criteria was initially created for Every Cure, a nonprofit focused on repurposing drugs for diseases with the high unmet medical need, the third method was to consider diseases previously identified as having a high unmet medical need within Every Cure, such as but not limited to ultra rare diseases and mental health disorders. This method identified diseases such as angiosarcoma, chordoma, undifferentiated pleomorphic sarcoma, depressive disorder, Duchenne muscular dystrophy, hidradenitis suppurativa (acne inversa), amyotrophic lateral sclerosis (ALS), and polycystic ovary syndrome.

The fourth method employed by the panel was to select a random assortment from an alphabetic list, to mitigate systematic biases in these first three selection criteria, to have increased exposure to less researched diseases and to select diseases with a lower perceived unmet medical need for the purpose of score generation. This method identified diseases such as basaloid follicular hamartoma, blue nevus, common wart, L-ferritin deficiency, and seasonal allergic rhinitis.

The final step involved reconciliation of these diseases from the four methods to ensure balanced spectra of disease characteristics of the final 40 diseases. For example, diseases selected to represent the spectrum of mortality: low-dermatitis, medium-breast cancer, high-ALS. With another example being the spectrum of disease modifying activity of a drug: low-achondroplasia, medium-depressive disorder, high-venous thromboembolism.

### Step 4: Quantitative criteria and scoring

We used objective scoring guidelines and a 5-point scale to categorize the results of each criterion, with higher scores indicating greater unmet medical need. We defined criteria by measurable terms (e.g., duration specified in weeks/months/years, prevalence rates per 100,000, etc.) and designed the scoring distributions for consistent interpretation and reproducibility by experts (S1–S3 Tables).

### Step 5: Expert scoring and reconciliation

Two medical experts (ES and NF) independently evaluated all diseases using any available resources, assigning scores for each criterion. A third medical expert (CB) helped reconcile differences greater than one point between the first two experts. The experts either reconciled one-point differences through discussion or accepted them as a reasonable margin of uncertainty. A variety of resources were employed on a disease dependent basis to arbitrate these discussions, and most frequently included PubMed articles such as disease summaries, clinical guidelines such as UpToDate, and disease databases such as Ophanet [9–11]. We randomly split the resulting dataset into a development set for LLM calibration (30 diseases), and an evaluation set to assess performance (10 diseases).

### Step 6: Prompt development and evaluating LLM performance

We iteratively refined prompts to generate consistent scores from LLMs, aiming for high accuracy compared to expert-derived scores. We used these criteria to evaluate the parity between expert and LLM scores rather than their relative rankings, as the latter required a reweighting step that occurred later in the process. Performance criteria included:

- Standard deviation within ±0.5 points.
- Mean difference within ±1 point.

Model parameters, including prompt formulation and temperature settings, were iterated on until outputs achieved results which scored within the acceptable evaluation metric thresholds. Once model performance was acceptable within evaluation metrics on the 30-question development set, we froze prompts and the model. We then ran these on our 10-question evaluation set to confirm performance on unseen data before running on all diseases.

### Step 7: Generation and evaluation of LLM outputs

We ran refined LLM prompts across all 22,701 diseases. We recorded the generation time per criterion to calculate the total generation time and recorded the cost per criterion and the total cost. To ensure the process remains accessible and reproducible, we avoided the use of fine-tuned models, retrieval-augmented generation (RAG), or other proprietary, nonpublic methods.

Before proceeding to downstream analysis, we manually audited the generated scores by using a random sampling approach to check for inaccuracies and verified the data types of all outputs to identify inappropriate values (e.g., noninteger values). Any inconsistent outputs were corrected via a single regeneration step using the initial prompt parameters.

### Step 8: Weighting of scoring factors

We calculated the final combined unmet medical need scores by taking weighted sums of scores. We assigned weights based on each criterion’s relative importance from first principles for Every Cure’s priorities; which is subjective for each organization and a sensitivity analysis is required if considering different weightings. For example, we gave current disease mortality a higher weight than cost considerations because Every Cure focuses on patient impact above all as a nonprofit organization. Organizations can further adjust these weights to align with their specific goals or prioritization strategies.

The resultant weighting criterion meant that the types of diseases being selected tended to be rare diseases without an effective standard of care, and resulting in high mortality/morbidity. We combined these weightings using the formula (Table 1):

**Table 1:** Weighting criteria are used to select for rare diseases without an effective standard of care, resulting in high mortality/morbidity.

Patient suffering	score weight
Commonality	0.25
Duration	0.5
QALYs lost	0.75
Mortality	1
**Standard of care**	**score weight**
Disease modification	1
Adverse events	0.75
Route of administration	0.5
Frequency of administration	0.25
**Accessibility**	**score weight**
Cost to patients	0.25
Robust supply	0.25
Regulatory barriers	0.25


Total score = (criteria 1* criteria 1 weight) + … + (criteria n * criteria n weight)

### Step 9: Evaluation of inter-rater agreement and thematic analysis

We assessed the inter-rater agreements between the LLM scores and expert scores using Spearman rank correlation and paired Wilcoxon signed-rank tests to assess for statistical significance on the pooled disease set (median paired difference = 0).

We further assessed LLM-derived scores for appropriateness for an Exploratory Factor Analysis (EFA) and Confirmatory Factor Analysis by conducting both Bartlett’s test of sphericity and the Kaiser-Meyer-Olkin (KMO) test.

### Step 10: Visualization of scoring distribution and example investigation

We created a visualization of the score distribution for each criterion by creating histograms of unmet medical need scores to identify deviations from expected patterns, aiding identification if any prompts needed further refinement.

## Findings

### Similarity between experts rankings

To determine inter-expert rater variability, we compared expert’s assessments across 440 scores (40 diseases * 11 criteria per disease). There were a total of 114 disagreements between the two experts who scored diseases independently from each other. Of these 114 disagreements, 90 (20.45%) were of ±1 point, 24 (5.45%) were of ±2 points, and no disagreements were of ≥3 points suggesting there was strong objectivity of the criteria devised (S4 Table). Disagreements of >1 point were mediated by the third expert to be within 1 point.

### Evaluation of LLM outputs and model choices

Foundation models and their parameters were evaluated based on the performance of their outputs compared to the expert rankings. We created 11 prompts, and the results of each prompt were evaluated by experts with metrics including standard deviation, mean difference, and disease-level deviation.

After testing across models, such as Open AI gpt-4o, gpt-3.5-turbo, gpt-4o-mini, the model which aligned closest to the medical expert scores on the development set was gpt-4o. We used gpt-4o as the model for all 11 prompts (S5 Table) [12].

All 11 prompts used a temperature of 0 to reduce sampling variability, aiming to reduce variability in scoring. We also used a system prompt which improved prompt stability and performance whilst improving the reliability of the output being in a consistent format:

*You are a medical doctor and Professor of Epidemiology trying to determine the unmet medical need for a disease. You wish to do this to determine the priority of diseases for research and development.*

*For each disease name, extract the following feature:*

*“{new_feature_name}: {feature_config[‘description’]}”.*

*Return ONLY a JSON object with the key “response” and the extracted values.*

*No explanations or other text.*

We found prompts written in the following style helped achieve performance metrics within the evaluation thresholds:

*Output only a number from 1 to 5; 1 = {criteria 1 name/ description}, 2 = {criteria 2 name/ description}, 3 = {criteria 3 name/ description}, 4 = {criteria 4 name/ description}, 5 = {criteria 5 name/ description}. Categorize the {concept of interest} for disease {disease name} into the above categories. {specific classification guidance based on errors with previous prompt iterations}.*

Prompts required a mean of 7.1 iterations or median of 6.5 iterations to achieve metrics within the evaluation set bound.

All but one prompt achieved a target mean difference of ±1 point compared to the expert scores in the development set (adverse events) (Tables 2 and 3). All prompts achieved the standard deviation difference target of ±0.5 points compared to the expert scores.

**Table 2:** The performance of diseases in the development set. Bold = not within target range.

Question	Expert mean	Expert std dev.	LLM mean	LLM std dev.	Mean difference	Std dev. difference
Commonality	2.83	1.44	3.00	1.11	−0.17	0.33
Duration	4.03	0.82	4.10	0.61	−0.07	0.21
QALYs lost	2.97	1.06	3.77	1.04	−0.80	0.02
Mortality	2.27	1.32	2.83	1.51	−0.57	−0.19
Disease modification	3.00	1.21	2.67	1.06	0.33	0.15
Adverse events	2.63	0.88	4.03	1.07	**−1.40**	−0.19
Administration route	2.77	1.16	2.57	1.63	0.20	−0.48
Frequency of administration	3.52	0.98	4.17	0.75	−0.65	0.23
Cost to patients	2.93	0.95	3.83	0.75	−0.90	0.21
Robust supply	2.42	1.08	3.33	0.99	−0.92	0.08
Regulatory barriers	1.40	0.77	2.00	1.02	−0.60	−0.25

**Table 3:** The performance of diseases in the evaluation set.

Question	Expert mean	Expert std dev.	LLM mean	LLM std dev.	Mean difference	Std dev. difference
Commonality	2.00	1.41	2.60	0.97	−0.60	0.45
Duration	4.30	1.25	3.60	1.17	0.70	0.08
QALYs lost	3.20	1.40	3.80	1.32	−0.60	0.08
Mortality	2.50	1.51	3.00	1.83	−0.50	−0.32
Disease modification	3.30	1.16	3.00	1.15	0.30	0.00
Adverse events	2.80	1.48	3.60	1.17	−0.80	0.30
Administration route	3.00	1.63	2.30	1.70	0.70	−0.07
Frequency of administration	4.20	1.40	3.90	1.20	0.30	0.20
Cost to patients	3.00	1.49	3.50	1.35	−0.50	0.14
Robust supply	2.90	1.60	2.90	1.10	0.00	0.49
Regulatory barriers	1.80	1.03	1.40	0.84	0.40	0.19

### Distribution of LLM unmet medical need scores

**Fig 1:**
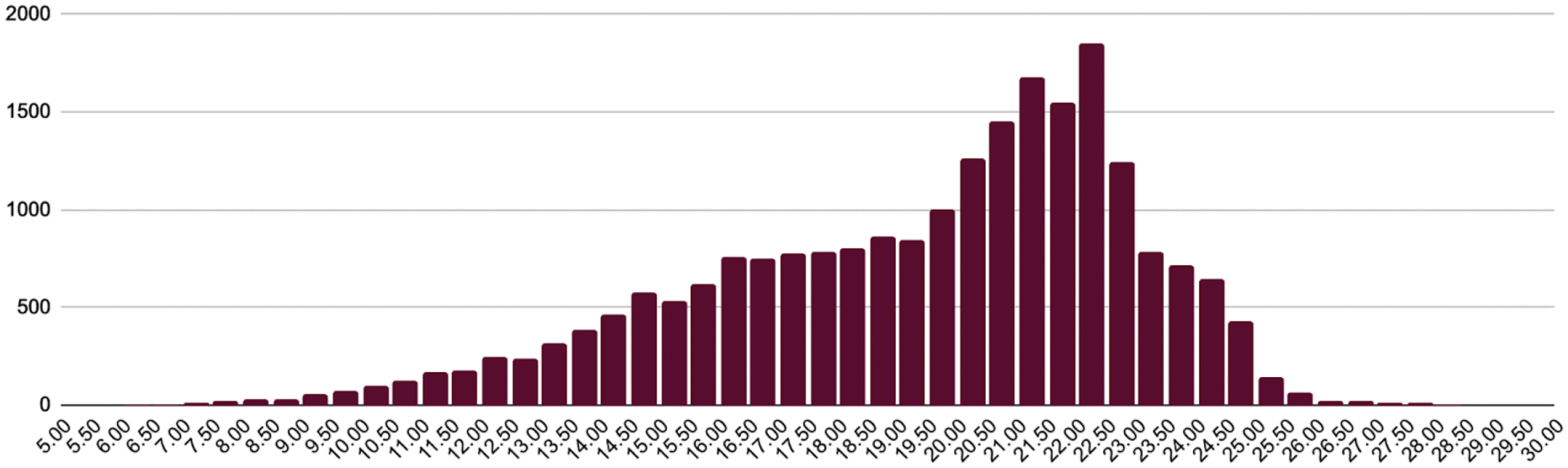
Histogram of weighted unmet medical need scores of 22,701 diseases.

Using the variable weightings reflecting the relative importance of each criterion for the purpose of identifying new treatments, the final distribution of scores is negatively skewed (skewness −0.69) (Fig 1).

The diseases contained within this distribution are a mixture of higher order disease groupings, disease subtypes, and semantic concepts in between. The balance of these terms likely creates the distribution seen.

### Generation cost analysis

Next, we sought to quantify the costs of this analysis. Since the input token cost of gpt-4o is $5.00 USD per 1 million tokens and $20.00 USD per 1 million output tokens through the OpenAI API in May 2025, the combined input and output token cost per prompt for 22,701 diseases was ~$10.00 USD [13]. There were additional ad hoc queries performed for testing that likely totaled <$10.00 USD, resulting in a combined total cost of ~$120.00 USD.

### Similarity between LLM and expert ratings

Next, we sought to assess the inter-rater agreement between LLM and expert ratings, which revealed notable alignment despite some discrepancies.

For each unmet medical need criterion, we assessed agreement between human and LLM ratings using rank correlation (Spearman), error metrics (mean absolute error/root mean square error), and bias detection (Wilcoxon signed-rank test) (Table 4). The Spearman correlation identifies whether the LLM preserves rank orderings; and the Wilcoxon test identifies whether it consistently over- or under-rates compared to humans. Spearman rank correlations were supplemented with bootstrap confidence intervals (1,000 resamples) to quantify uncertainty in rank correlation estimates between human and LLM ratings.

**Table 4:** Spearman rank correlation, confidence intervals, errors, and Wilcoxon signed-rank across scoring criteria.

Question	Spearman rank correlation	Spearman rank 95% CI	Mean absolute error (MAE)	Root mean square error (RSME)	Wilcoxon signed-rank
Commonality	0.653	0.421,0.816	0.825	1.129	0.158
Duration	0.440	0.107,0.712	0.475	0.798	0.193
QALYs lost	0.822	0.693,0.902	0.850	1.031	0.000
Mortality	0.845	0.724,0.925	0.775	1.090	0.001
Disease modification	0.037	(−0.296),0.360	1.375	1.677	0.131
Adverse events	0.674	0.450,0.828	1.400	1.585	0.000
Administration route	0.737	0.547,0.870	0.750	1.124	0.079
Frequency of administration	0.045	(−0.3710), 0.403	0.838	1.170	0.034
Cost to patients	0.653	0.411,0.821	0.850	1.146	0.000
Robust supply	0.231	(−0.126),0.570	1.163	1.490	0.004
Regulatory barriers	−0.084	(−0.389),0.223	1.000	1.396	0.113

Several criteria show strong Spearman rank correlation between human rankings and LLM-based rankings, indicating agreement in relative prioritization across diseases, such as mortality, ρ = 0.845 (95% CI [0.724, 0.925]); QALYs lost, ρ = 0.822 (95% CI [0.693, 0.902]), and route of administration: ρ = 0.737 (95% CI [0.546, 0.874]).

These suggest that LLMs capture the same general ranking trends as human experts for these high-impact clinical and treatment burden dimensions.

For other criteria, Wilcoxon signed-ranked did not detect a significant difference in median between the LLM and expert scores for these criteria (paired test; *n* = 40), meaning LLMs and humans are not only ranking items similarly but also assigning similar absolute scores (equivalence was not established due to small sample size). Such as commonality (*p* = 0.1583); duration (*p* = 0.1927); and disease modification (*p* = 0.1309).

This is particularly important for commonality and route of administration, where both the rank and magnitude agree, strengthening confidence in LLM performance on these dimensions.

Although likely underpowered due to small sample sizes, the assessment described shows that no criterion showed strong disagreement across both tests (i.e., low correlation and significant difference), suggesting that even when LLMs diverge from expert scores, they do so in either scale or ordering, but not both. Therefore, we considered LLM scores to be suitable for our use case.

### Thematic analysis through factor analysis

To examine the themes and underlying dimensional structure of LLM generated scores, we aimed to complete an Exploratory Factor Analysis (EFA). Before doing so, we first evaluated whether the LLM-derived scores were appropriate for EFA by conducting both Bartlett’s test of sphericity and the KMO test of sampling adequacy (Table 5).

**Table 5:** Kaiser-Meyer-Olkin measure sampling accuracy across all criteria.

Criteria	Measure sampling accuracy (MSA)
Commonality	0.73
Duration	0.74
QALYs lost	0.68
Mortality	0.84
Disease modification	0.81
Adverse events	0.89
Route of administration	0.82
Frequency of administration	0.77
Cost to patients	0.89
Robust supply	0.79
Regulatory barriers	0.82
**overall**	**0.81**

Bartlett’s Test Chi-squared = 329.28, df = 55, *p* < 1e-40. This highly significant result confirms that the correlation matrix is not an identity matrix (where variables are uncorrelated), indicating sufficient inter-item correlation for factor analysis. KMO Test Overall measure sampling accuracy (MSA) = 0.81, considered “meritorious”. All individual items had MSA > 0.68 (desired outcome is > 0.6). These values support the sampling adequacy and justify proceeding with EFA.

**Fig 2:**
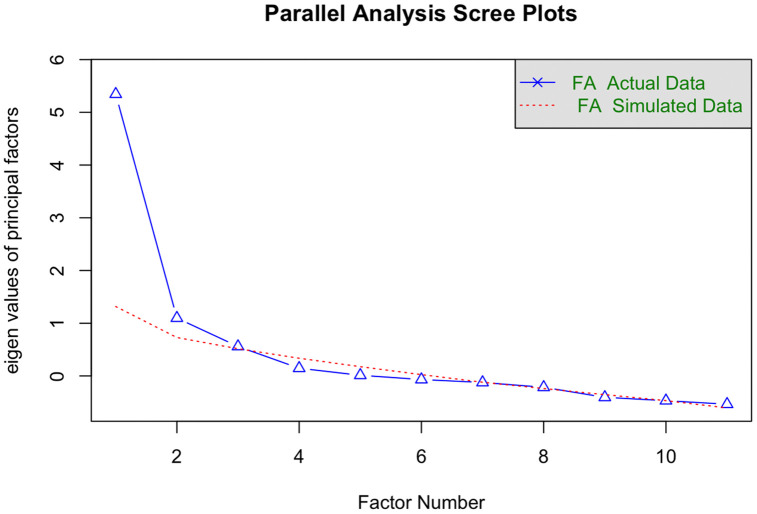
Parallel Analysis Scree Plot for values of principal factors against simulated data.

A parallel analysis suggests that the number of factors is 4 and the number of components is not applicable (Fig 2).

**Fig 3:**
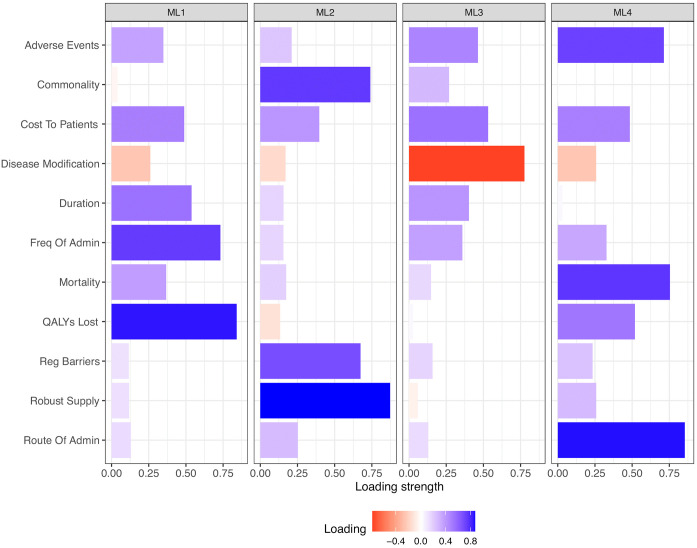
Exploratory factor analysis groupings based on loading strength.

Criteria were grouped in four groups from the factor analysis, based on highest loadings (Fig 3).

### ML1 - Disease Burden:

Reflects overall clinical and personal burden of disease, incorporating severity, mortality, treatment frequency, and financial cost

- QALYs lost (0.841)
- Frequency of admin (0.732)
- Adverse events (0.348)

### ML2 - Structural and Access Barriers:

Indicates logistical/system-level challenges in treatment availability and regulation

- Commonality (0.740)
- Robust supply (0.873)
- Regulatory barriers (0.674)

### ML3 - Disease Modifiability and Chronicity:

Reflects aspects of long-term management, cost, and (inversely) the extent to which conditions can be modified or cured

- Disease_modification (–0.776) (negative loading)
- Cost to patients (0.531)
- Duration (0.403)

### ML4 - Treatment complexity:

Represents complexity and intensity of treatment delivery, with additional loadings from severe outcome indicators

- Route of administration (0.854)
- Adverse events (0.713)
- Mortality (0.754)

The model replicates many latent structures seen in EFA (e.g., separation of clinical, systemic, and treatment factors). However, overall model fit is poor, largely due to a small sample size (*n* = 40), which is underpowered for confirmatory factor analysis (CFA) with some items showing cross-loadings or weak structure.

### Evaluation of scores across illustrative diseases and within related diseases

Given that some diseases have similar pathophysiology and similar names, but different standards of care and therefore different unmet medical needs, we qualitatively assessed some related diseases to evaluate the relative precision of the scores.

For parkinsonism, Parkinson disease has several on-label treatment options such as levodopa, dopamine agonists, MAO-B inhibitors, and device therapies (deep brain stimulation) [14]. Whereas multiple system atrophy and progressive supranuclear palsy (PSP) only have symptomatic off-label use of levodopa. Given this, multiple system atrophy and PSP would be expected to have a greater unmet medical need score than Parkinson disease and this is the case (Table 6).

**Table 6:** Total weighted scores of diseases with different treatments but related conditions.

Type	Disease ID	Disease Name	Total weighted score
Parkinsonism	MONDO:0007803	Multiple system atrophy	23.25
	MONDO:0019037	Progressive supranuclear palsy	23.00
	MONDO:0005180	Parkinson disease	18.50
Multiple sclerosis	MONDO:0000450	Secondary progressive multiple sclerosis	22.50
	MONDO:0000451	Primary progressive multiple sclerosis	22.50
	MONDO:0000452	Progressive relapsing multiple sclerosis	22.25
	MONDO:0005314	Relapsing-remitting multiple sclerosis	18.50
Gaucher disease	MONDO:0009266	Gaucher disease type II	22.75
	MONDO:0009267	Gaucher disease type III	21.00
	MONDO:0009265	Gaucher disease type I	17.50

For multiple sclerosis, there has been more development of disease modifying therapies for relapsing-remitting multiple sclerosis (RRMS), such as injectable interferons, glatiramer acetate, oral agents, and monoclonal antibodies, compared to other multiple sclerosis subtypes [15]. As a result, the RRMS subtype of multiple sclerosis would be expected to have a lower unmet medical need score than other subtypes and this is also the case (Table 6).

In Gaucher disease type 1 there are effective enzyme-replacement therapies and substrate-reduction agents which treat the disease [16]. In type 2 and 3, there are no approved therapies, which would be expected to result in a higher unmet medical need score for these subtypes, which is the case (Table 6).

During Every Cure’s comprehensive “all drugs versus all diseases” reviews, the Medical Team rapidly shifts focus across hundreds of diseases, each with its own complex characteristics and clinical context. With over 22,000 diseases to evaluate, it becomes challenging to maintain a clear sense of the relative burden and suffering caused by each, especially when many are rare and unfamiliar with differences in the type of unmet medical need encountered by each patient. To support consistent and evidence-based decision-making during this process, the team considers a disease’s relative ranking within the unmet medical need score. This score serves as one of several decision aids, allowing the team to approximate how the unmet medical need of one disease compares to another and to discern its absolute position within the broader set of all diseases under review.

For example, understanding the unmet medical need of eponymous syndromes without aids requires memorization as they rarely contain any descriptive terms. Timothy syndrome (MONDO:0010979) and Lown-Ganong-Levine syndrome (MONDO:0007174) are both rare diseases with an estimated prevalence of <1 in 1 million and both have eponymous names [17,18].

However, patients with Timothy syndrome experience substantially greater suffering than those with Lown-Ganong-Levine syndrome. Timothy syndrome is a severe multisystem genetic disorder caused by CACNA1C mutations, leading to life-threatening cardiac arrhythmias, congenital heart defects, developmental delay, autism-spectrum features, immune dysfunction, and other systemic complications. The disease manifests early, often in infancy, and is associated with high mortality and significant disability among survivors [17]. In contrast, Lown-Ganong-Levine syndrome is primarily a cardiac conduction abnormality that causes episodes of supraventricular tachycardia but is frequently asymptomatic or manageable with medication or ablation. It does not typically affect other organ systems or life expectancy [18].

This difference in suffering is reflected in the scores for both diseases, with the score of Timothy syndrome being 24.25 (rank 959 - top 4.2% of scores) and Lown-Ganong-Levine syndrome being 11.75 (rank 21,935 - top 96.7% of scores). This example demonstrates how these scores can enhance the speed and quality of experts’ assessments.

## Discussion

This is a systematic methodology leveraging LLMs to quantify unmet medical needs across diverse and heterogeneous diseases. This approach mitigates previous challenges in defining what to research, including incomplete data representation, ontological mapping difficulties, and variability in defining standard-of-care treatments. Utilizing the knowledge corpus of LLMs, these challenges have been circumvented, and an accessible, scalable, and maintainable assessment tool has been made.

The implementation of a standardized, systematic method for generating unmet medical need scores offers benefits for the development of treatments for rare diseases. It enables objective prioritization, whilst using weighting of scores to remain flexible to each organization’s unique needs, ensuring that research and development efforts focus on conditions with the highest unmet medical needs. This systematic approach facilitates consistent assessments across various diseases and populations, improving the comparability of findings and supporting evidence-based decision-making. The reproducibility of this methodology allows for the integration of new data as it becomes available, maintaining the relevance and accuracy of assessments. This adaptability is particularly crucial in the context of rare diseases, where emerging information can substantially influence the understanding of disease burden and treatment efficacy.

In this application of the method, we adjusted the weighting of factors to score highly for rare diseases without an effective standard of care, resulting in high mortality/morbidity for patients, which is aligned with our strategic goals as an organization.

The distribution of unmet medical need scores is approximately what was expected as most diseases do not have safe or effective disease modifying treatments, which creates a positive skew. Combined with the inclusion of higher order disease groupings this will flatten the unmet medical need of diseases and their subtypes, resulting in a negative skew to the distribution. The balance of these factors is likely what is responsible for the shape of the distribution observed.

Our approach has several limitations. We aimed for a framework that is mutually exclusive and collectively exhaustive to define unmet medical need but despite this, some overlap persists. For example, disease duration and QALYs lost are naturally correlated, as longer illnesses often result in greater total QALY loss. Nevertheless, these criteria capture important dimensions of need: the chronological span of the condition versus the intensity of a patient’s suffering.

Whilst LLMs have an increased risk of hallucination when there is limited data for a specific disease, we have found through both evaluation and use of this database by our Medical Team that the combination of iterative prompt development and multiple criteria scoring of any one disease means that pragmatically, the scores are useful to bridge gaps in information between databases, although confidence in such outputs should be treated with caution. However, because the LLM outputs are flattened into a single integer to create a score, the underlying reasoning for each step remains challenging to interpret. This flattening makes it difficult to isolate the impact of specific hallucinations or poorly founded outputs, such as considering the first few words only in a long disease name, without introducing subjective investigator bias during the assessment process. Those wishing to reproduce this method should consider the cost of adding increasing technical complexity with the improved ability to interrogate each output by adding an intermediate reasoning step before score generation.

The impact of potential LLMs hallucinations were mitigated by utilizing a composite score from 11 different prompts which reduces the relative impact of any potentially incorrect individual score. LLMs also tend to struggle with long disease names which are quite descriptive and sometimes provide scores for part of this disease name (e.g., MONDO:0011048 “epilepsy-microcephaly-skeletal dysplasia syndrome” is prone to returning values for “epilepsy”) [19]. Although these examples were infrequent, we mitigated this by using prompt engineering and disease synonyms in the input query to improve the LLM output recognition of the entire disease name being the term to score. To further mitigate hallucinations, the framework could incorporate structured biomedical ontologies, such as the Human Phenotype Ontology (HPO), as an intermediate validation step. This step might ensure that the disease being evaluated is accurately identified and that its phenotypic characterization aligns with established clinical standards before the final score is generated [20]. Implementing an intermediate reasoning step or a retrospective LLM justification would further enhance the auditability of the scores by ensuring they are well-founded. This could further be expanded by creating an intermediate confidence scoring assessment.

The use of LLMs has the potential to introduce epistemic circularity because the LLMs are pretrained on biomedical text that encodes disease characteristics such as prevalence and treatment standards of care which have the tendency to reflect preexisting health priorities rather than being a truly independent quantification of unmet medical need. An advantage of this method is the ability to reweight individual dimensions of unmet medical need, which partially mitigates the influence of preexisting health priorities. By adjusting these weights, we can partially counteract the bias toward high-prevalence diseases inherent in the Global Burden of Disease classification, allowing us to prioritize characteristics like high mortality instead. There are methods which could be considered to further mitigate the impact of knowledge circularity through less literature-dependent signals, such as incorporating epidemiological registries or orphan disease datasets. These could be incorporated using the data itself or via model context protocols (MCPs) with the help of LLMs, acknowledging that the latter will likely be faster but also subject to data leakage from pretraining sources.

While our methodology aims for objectivity, a literature-based approach inevitably reflects the geographic and socioeconomic biases of current research. We use objective criteria and reweighting to minimize this impact; however, those looking to highlight under-represented conditions should consider supplementing this approach with further adjustments, such as population-based weighting. We randomly split the 40 diseases into development and evaluation sets to minimize selection bias; however, this decision meant the development set might have been less balanced than the total pool of diseases.

When considering which specific foundation model to use, each model involves making trade-offs between cost, parameter size, and infrastructure ease-of-use in addition to other considerations. Due to the deconstructionist approach to create this score, by taking an abstract problem and breaking it into more concrete tasks, we found that the choice of model did not meaningfully influence performance for our use case during testing, though comprehensive benchmarking across models remains future work. Groups intending to use this method should consider which model is most appropriate for their problem but will likely benefit more from dedicating their efforts on accurately evaluating output performance. Given the rapid pace of foundation model releases, we encourage those replicating this method to consult current performance benchmarks, such as the LM Arena, to select the most capable models available at the time of their analysis [21].

LLM cost values are approximate rather than exact because the 11 fields were generated in rapid succession to one another, meaning it is difficult to attach a precise cost to each field. The small sample size of these sets meant that development and testing were fast, but at the potential cost of stability and generalizability. A larger validation set (e.g., > 100 diseases) would provide more reliable performance estimates in future work. The adverse events prompt did not achieve the mean difference target of ±1 point so will be redesigned in the future versions to ensure it reaches this target range. Adverse events likely did not achieve the mean difference target because the LLMs might have been considering the adverse events of the diseases, rather than the adverse events of the standards of care for those diseases. Whilst not materially affecting the final unmet medical need scores, the small size meant that the results were underpowered for a confirmatory factor analysis. This could be improved by using larger sample sizes (e.g., ≥100) or considering alternative model structures or bi-factor models.

We now use the unmet medical need score quantitatively to compare potential impact to patients of the most promising repurposing opportunities. Unmet medical need represents a distinct and important orthogonal perspective to mechanistic treatment predictions. For instance, we integrate these scores into our recommendation algorithm to prioritize treatments for diseases with high unmet medical needs, which are then surfaced to the Every Cure Medical Team for assessment. These scores also guide our resource allocation for computationally intensive drug-disease analyses, ensuring we focus our efforts and resources where they can achieve the maximum patient impact.

This accessible, scalable methodology enables funders and researchers, across governments, universities, healthcare organizations, and disease groups to tailor prioritization efforts according to their organizational objectives. This method creates a tool which is practical and transparent in its inputs (criteria/weights), with accuracy supported by initial agreement testing on 40 diseases to streamline research prioritization.

## Supporting information

S1 Table Four questions to quantify patient suffering in unmet medical need and their scoring criteria. (DOCX)

S2 Table Four questions to quantify the standard of care of treatment in unmet medical need and their scoring criteria. (DOCX)

S3 Table Three questions to quantify the accessibility of treatment in unmet medical need and their scoring criteria. (DOCX)

S4 Table Disagreements between two independent experts before meditation by a third expert to resolve disagreements >1 point. (DOCX)

S5 Table All 11 prompts used to generate unmet need scores across diseases. The prompt contents at the end of each prompt were added to correct for systematic biases in outputs during development. (DOCX)

## Abbreviations

AI: artificial intelligence
ALS: amyotrophic lateral sclerosis
CFA: confirmatory factor analysis
COPD: chronic obstructive pulmonary disease
EFA: exploratory factor analysis
HPO: human phenotype ontology
KMO: Kaiser-Meyer-Olkin
LLMs: large language models
MCPs: model context protocols
MSA: measure sampling accuracy
PSP: progressive supranuclear palsy
QALYs: quality adjusted life years
RAG: retrieval-augmented generation
RRMS: relapsing-remitting multiple sclerosis.
