# Correction: Quantify unmet medical need across the disease landscape – A large language model-based methodology

**DOI:** 10.1371/journal.pmed.1005048

**Published:** 2026-04-10

**Authors:** Elliott W. Sharp, Nicholas Fragola, Charlotte Blewitt, Matthew Goddeeris, Lee Lancashire, Charlie Hempstead, David C. Fajgenbaum

The word “Quantifying” is misspelled in the article title. The correct title is: Quantifying unmet medical need across the disease landscape – A large language model-based methodology. The correct citation is: Sharp EW, Fragola N, Blewitt C, Goddeeris M, Lancashire L, Hempstead C, et al. (2026) Quantifying unmet medical need across the disease landscape – A large language model-based methodology. PLoS Med 23(3): e1004798. https://doi.org/10.1371/journal.pmed.1004798.
