# Supplementary material for: Quantify unmet medical need across the disease landscape – A large language model-based methodology
Source: PLoS Med. 2026 Mar 12;23(3):e1004798. doi: 10.1371/journal.pmed.1004798 (PMC12981509; doi:10.1371/journal.pmed.1004798)
Supplement: S1 Table — (DOCX) [file pmed.1004798.s001.docx]

| **Patient suffering** | **Detailed criteria** | **Score** |
| --- | --- | --- |
| **How common is the disease?** | | |
| Very common: | For chronic diseases use prevalence and for acute diseases use incidence. If chronic, prevalence ≥1% of the population with broad global and demographic impact. If acute, incidence >1,000 cases per 100,000 people per year. | 1 |
| Common: | For chronic diseases use prevalence and for acute diseases use incidence. If chronic, prevalence 0.1-1% of the population (1 in 1,000 to 1 in 100) with broad global impact. If acute, incidence between 100 and 1,000 cases per 100,000 people per year. | 2 |
| Intermediate: | For chronic diseases use prevalence and for acute diseases use incidence. If chronic, prevalence 0.01-0.1% of the population (1 in 10,000 to 1 in 1,000). If acute, incidence between 10 to 100 cases per 100,000 people per year. | 3 |
| Rare: | For chronic diseases use prevalence and for acute diseases use incidence. If chronic, prevalence 0.01-0.001% of the population (1 in 10,000 to 1 in 100,000). If acute, incidence between 1 to 10 cases per 100,000 per year. | 4 |
| Very rare: | For chronic diseases use prevalence and for acute diseases use incidence. If chronic, prevalence <0.001% of the population (fewer than 1 in 100,000). If acute, incidence <1 case per 100,000 per year. | 5 |
| **What is the duration of the disease?** | | |
| Acute: | Diseases lasting days, which are often self-limiting (e.g., food poisoning). | 1 |
| Sub-acute: | Diseases lasting weeks, which are sometimes self-limiting (e.g., broken arm). | 2 |
| Intermediate: | Diseases lasting months, almost always requiring treatments (e.g., reactive arthritis). | 3 |
| Chronic: | Diseases lasting years, almost always requiring treatments (e.g., Bell’s Palsy). | 4 |
| Lifelong: | Diseases persisting for a lifetime, almost always requiring treatments (e.g., type 1 diabetes mellitus). | 5 |
| **Patient QALYs lost from the disease** | | |
| Very low morbidity: | Disease has minimal impact on activities of daily living, usually in vulnerable populations. | 1 |
| Low morbidity: | Disease has some impact on activities of daily living, in widespread populations. | 2 |
| Moderate morbidity: | Disease has a moderate impact on activities of daily living. | 3 |
| High morbidity: | Disease associated with high impact on activities of daily living. | 4 |
| Very high morbidity: | Disease has a very high impact on activities of daily living (e.g., patients are bedbound). | 5 |
| **What is the 5-year survival rate?** | | |
| No mortality: | No appreciable mortality. | 1 |
| Low mortality: | >80% 5-year survival rate. | 2 |
| Moderate mortality: | 50-80% 5-year survival rate. | 3 |
| High mortality: | 20-50% 5-year survival rate. | 4 |
| Very high mortality: | <20% 5-year survival rate. | 5 |

**S1 Table. Four questions to quantify patient suffering in unmet medical need and their scoring criteria.**
