# Supplementary material for: Quantify unmet medical need across the disease landscape – A large language model-based methodology
Source: PLoS Med. 2026 Mar 12;23(3):e1004798. doi: 10.1371/journal.pmed.1004798 (PMC12981509; doi:10.1371/journal.pmed.1004798)
Supplement: S2 Table — (DOCX) [file pmed.1004798.s002.docx]

| **Standard of care** | **Detailed criteria** | **Score** |
| --- | --- | --- |
| **What disease modification does the current standard of care provide?** | | |
| Curative / fully preventative: | Standard of care results in no measurable disease, with effectively no chance of relapse. | 1 |
| High disease modification: | Standard of care causes full remission or disease control with no persisting impact on quality of life or activities of daily living (e.g., insulin for type 1 diabetes mellitus). | 2 |
| Moderate disease modification: | Standard of care results in improvement in morbidity and / or mortality (i.e., increased life expectancy by years). | 3 |
| Low disease modification: | Standard of care results in partial improvement in morbidity and / or mortality (i.e., better than doing nothing). | 4 |
| Symptom relief only: | Standard of care provides no disease modifying activity and only aims to alleviate symptoms of the disease (e.g., analgesics for pain relief, inhalers for shortness of breath). | 5 |
| **What are the side effects / adverse events associated with the current standard of care?** | | |
| Grade 1: | Standard of care produces side effects with mild symptoms, asymptomatic or only observed through clinical or diagnostic findings, no intervention needed. | 1 |
| Grade 2: | Standard of care produces side effects with moderate symptoms, minimal intervention required, may limit some activities of daily living. | 2 |
| Grade 3: | Standard of care produces side effects with severe, medically significant, may require hospitalization or interfere with activities of daily living. | 3 |
| Grade 4: | Standard of care produces side effects which can be life-threatening, often requiring urgent interventions. | 4 |
| Grade 5: | Standard of care produces side effects which might result in death. | 5 |
| **What is the route of administration for the current standard of care?** | | |
| Over counter oral: | Available without prescription and widely available at local pharmacies or stores. | 1 |
| Oral prescription / other: | Requires a prescription but is widely available at local pharmacies or stores. | 2 |
| Intravenous / injection / behavioral: | Requires travel to a medical facility (e.g., therapy, intravenous infusions) or self-administered at-home injections. | 3 |
| Minor surgical procedure: | Requires local but not general anesthetic, often an outpatient procedure (e.g., excision of a skin lesion). | 4 |
| Major surgical procedure: | Requires general anesthetic (e.g., abdominal operation). | 5 |
| **What is the frequency of administration / monitoring required for the current standard of care?** | | |
| Once only: | e.g., surgery | 1 |
| Once per month (or less): | e.g., monthly depot injections, infrequent therapy interventions | 2 |
| Once per week: | e.g., intravenous infusions, behavioral interventions | 3 |
| Once per day: | e.g., oral medications / self-administered injections | 4 |
| Multiple times per day: | e.g., oral medications | 5 |

**S2 Table. Four questions to quantify the standard of care of treatment in unmet medical need and their scoring criteria.**
