# Supplementary material for: Quantify unmet medical need across the disease landscape – A large language model-based methodology
Source: PLoS Med. 2026 Mar 12;23(3):e1004798. doi: 10.1371/journal.pmed.1004798 (PMC12981509; doi:10.1371/journal.pmed.1004798)
Supplement: S3 Table — (DOCX) [file pmed.1004798.s003.docx]

| **Accessibility** | **Detailed criteria** | **Score** |
| --- | --- | --- |
| **What is the cost to patients of the current standard of care?** | | |
| Very low: | $10-$100 per month (e.g., many over the counter medicines, aspirin, ibuprofen). | 1 |
| Low: | $100-$1,000 per month (e.g., many generic prescription medicines, bisoprolol, warfarin). | 2 |
| Moderate: | $1,000-$10,000 per month (e.g., many patented small molecule medicines, small out-patient procedures, some behavioral interventions). | 3 |
| High: | $10,000-$100,000 per month (e.g., many patented biologics; in-patient procedures under general anesthetic). | 4 |
| Very high: | >$100,000 per month (e.g. some patented biologics; gene therapy). | 5 |
| **How robust is the supply chain for the current standard of care?** | | |
| Very high: | Available worldwide, few storage concerns, long shelf-life. | 1 |
| High: | Available worldwide, occasional availability issues. | 2 |
| Moderate: | Only available in some countries but a robust supply chain in these countries OR needs a cold chain. | 3 |
| Low: | Only available in some countries, several manufacturers but reliability issues with occasional stock-outs. | 4 |
| Very low: | Only available in very few countries with a single manufacturer and an unreliable supply chain with frequent stock-outs. | 5 |
| **What are the regulatory barriers of the current standard of care?** | | |
| Low: | Standard of care is licensed to treat the disease in at least three stringent regulatory authority regions (e.g., Food and Drug Administration in the United States of America, European Medicines Agency in Europe). | 1 |
| Not applicable | No score available. | 2 |
| Moderate: | Standard of care is licensed to treat the disease in only one stringent regulatory authority region (e.g. FDA = USA). | 3 |
| Not applicable | No score available. | 4 |
| High: | Standard of care is not licensed to treat the disease in any stringent regulatory authority region and is only used off-label OR available on-label by at least one non-stringent regulatory authority (e.g., China, India, Russia). | 5 |

**S3 Table. Three questions to quantify the accessibility of treatment in unmet medical need and their scoring criteria.**
