# Supplementary material for: Quantify unmet medical need across the disease landscape – A large language model-based methodology
Source: PLoS Med. 2026 Mar 12;23(3):e1004798. doi: 10.1371/journal.pmed.1004798 (PMC12981509; doi:10.1371/journal.pmed.1004798)
Supplement: S4 Table — (DOCX) [file pmed.1004798.s004.docx]

|  | **Patient suffering** |  |  |  | **Standard of care** |  |  |  | **Access.** |  |  |
| --- | --- | --- | --- | --- | --- | --- | --- | --- | --- | --- | --- |
| **Disease** | **prevalence** | **duration** | **QALYs lost** | **mortality** | **disease modification** | **adverse events** | **route of admin.** | **freq. of admin.** | **cost to patients** | **robust supply** | **regulatory barriers** |
| Achondroplasia | 0 | 0 | 2 | 0 | 1 | -1 | 0 | -2 | -2 | -2 | -1 |
| acne inversa, familial, 1 | 0 | 1 | 2 | 0 | 0 | 0 | 0 | 0 | 1 | 0 | 0 |
| acute myeloid leukemia | -1 | 0 | 0 | 1 | -1 | -1 | -2 | -1 | 0 | 0 | 0 |
| amyotrophic lateral sclerosis | 0 | 1 | 0 | -1 | 0 | 1 | 0 | 0 | 1 | 0 | 2 |
| angiosarcoma | 0 | 0 | 1 | 0 | 0 | 0 | -1 | 0 | 0 | 0 | 2 |
| asthma | 0 | 0 | 0 | 0 | 0 | 0 | 0 | 0 | 0 | 0 | 0 |
| autism spectrum disorder | 0 | 0 | 1 | 0 | -1 | 0 | 0 | 0 | 0 | 2 | 0 |
| basaloid follicular hamartoma | 0 | -1 | 0 | 0 | 0 | 1 | 0 | 0 | 0 | 0 | 0 |
| blue nevus | 0 | 0 | 0 | 0 | 0 | 0 | 0 | 0 | 0 | 0 | 0 |
| brain hypoxia - ischemia | -1 | -1 | 1 | 0 | 0 | 0 | -1 | 1 | 0 | 0 | 0 |
| breast cancer | -1 | -1 | 0 | 1 | -1 | -1 | 0 | 0 | -1 | -1 | 0 |
| Chordoma | 0 | -1 | 1 | 0 | -1 | 0 | 0 | 0 | -1 | 0 | 0 |
| chronic obstructive pulmonary disease | 0 | 0 | 0 | 0 | 0 | 0 | 0 | 0 | 0 | 0 | 0 |
| common wart | 0 | 0 | 0 | 0 | 0 | 0 | 0 | 0 | 0 | 0 | 0 |
| coronary microvascular disorder | -2 | 1 | 0 | 0 | -1 | 0 | 0 | 0 | 0 | 0 | 0 |
| dementia | -2 | 1 | 0 | -1 | -1 | 0 | 0 | 1 | 0 | 1 | 0 |
| depressive disorder | -1 | 0 | 1 | 0 | 0 | 0 | 0 | 0 | 0 | 1 | 0 |
| dermatitis | 0 | 0 | 1 | 0 | 0 | 0 | 0 | 0 | 0 | 0 | 0 |
| developmental and epileptic encephalopathy | 0 | 0 | 0 | 0 | 0 | 0 | 0 | 0 | 0 | 0 | 0 |
| Duchenne muscular dystrophy | 0 | 0 | 0 | 0 | 0 | 0 | 0 | 0 | 0 | 0 | 0 |
| familial hypercholesterolemia | 0 | 0 | 0 | 0 | 0 | 0 | 0 | 0 | 0 | 0 | 0 |
| fibromyalgia | 0 | 0 | 0 | 0 | -1 | 1 | 1 | -2 | 1 | 1 | 0 |
| generalized anxiety disorder | 0 | 0 | 2 | 0 | 0 | 0 | 0 | 0 | 0 | 0 | 0 |
| glioblastoma | 0 | 0 | 0 | 0 | 0 | 0 | 0 | 0 | 0 | 0 | 0 |
| mucocutaneous lymph node syndrome | 0 | 0 | 1 | 0 | 0 | 0 | 0 | 0 | -1 | -1 | 0 |
| L-ferritin deficiency | 0 | -2 | 0 | -1 | 0 | 0 | 0 | 1 | 0 | 0 | 0 |
| limb-girdle muscular dystrophy | 0 | 0 | 1 | 0 | 1 | 0 | 0 | -1 | 0 | 1 | 0 |
| long COVID-19 | 2 | 1 | 1 | 0 | 0 | 0 | 1 | -1 | 0 | 0 | 0 |
| NAFLD1 | 0 | 0 | 0 | 0 | 0 | 0 | 0 | 0 | 0 | 0 | 0 |
| osteoarthritis, knee | 0 | 0 | 0 | 0 | 0 | 0 | 0 | 0 | 0 | 0 | 0 |
| pancreatic ductal adenocarcinoma | 0 | 0 | 0 | 0 | 0 | 0 | 0 | 0 | 0 | 0 | 0 |
| polycystic ovary syndrome | -1 | 1 | 1 | 0 | -2 | -1 | 0 | 0 | 0 | 1 | 0 |
| prostate adenocarcinoma | 0 | 0 | 0 | 0 | 0 | 0 | 0 | 0 | 0 | 0 | 0 |
| seasonal allergic rhinitis | 0 | 0 | 0 | 0 | 0 | 0 | 0 | 0 | 0 | 0 | 0 |
| sickle cell anemia | -2 | 0 | 0 | -1 | 0 | 0 | 2 | 0 | 1 | 2 | 2 |
| stroke disorder | -1 | -1 | 1 | -1 | 0 | 0 | -1 | 1 | 0 | 0 | 0 |
| type 2 diabetes mellitus | 0 | 0 | 1 | 1 | 0 | -1 | 1 | 2 | -1 | -2 | 0 |
| undifferentiated pleomorphic sarcoma | 0 | -1 | 1 | 1 | 0 | 0 | 0 | 0 | 0 | 0 | 0 |
| vascular cancer | 0 | 0 | 0 | 0 | 0 | 0 | 0 | 0 | 0 | 0 | 0 |
| venous thromboembolism | -1 | 1 | 1 | 0 | 0 | 0 | -2 | 0 | -2 | -1 | 0 |

**S4 Table. Disagreements between two independent experts before meditation by a third expert to resolve disagreements >1 point.**
