# Supplementary material for: Quantify unmet medical need across the disease landscape – A large language model-based methodology
Source: PLoS Med. 2026 Mar 12;23(3):e1004798. doi: 10.1371/journal.pmed.1004798 (PMC12981509; doi:10.1371/journal.pmed.1004798)
Supplement: S5 Table — (DOCX) [file pmed.1004798.s005.docx]

| **Criteria** | **Prompt** |
| --- | --- |
| Patient suffering | |
| Commonality | Categorize the commonality of "{disease}" {or disease synonyms} into the one of the following ordinal categories:  - 1 = very common  - 2 = common  - 3 = intermediate  - 4 = rare  - 5 = very rare  Utilize prevalence for chronic diseases and incidence for acute diseases.  Very common diseases should affect more than 1% of the global population.  Very rare diseases should have an incidence of less than 1 in 100,000.  Where multiple diseases are listed, use only the first. |
| Duration | Categorize the duration of "{disease}" {or disease synonyms} post-accurate diagnosis into the one of the following ordinal categories:  - 1 = acute (days)  - 2 = sub-acute (weeks)  - 3 = intermediate (months)  - 4 = chronic (years)  - 5 = lifelong  Deduct points for diseases with a treatment or standard of care that can quickly resolve the condition. |
| QALYs lost | Categorize the quality of life adjusted years (QALYs) lost of "{disease}" {or disease synonyms} into one of the following ordinal categories based on the impact that the disease has on activities of daily living (ADLs):  - 1 = very low morbidity  - 2 = low morbidity  - 3 = moderate morbidity  - 4 = high morbidity  - 5 = very high morbidity  If the disease only has impact on ADLs in vulnerable populations, score 1. |
| Mortality | Categorize the mortality of "{disease}" {or disease synonyms} into one of the following ordinal categories based on the 5-year survival rate:  - 1 = no mortality  - 2 = low mortality  - 3 = moderate mortality  - 4 = high mortality  - 5 = very high mortality  For diseases with no appreciable mortality, score 1. For diseases that result in death in 80% of cases, score 5. Add a point if the disease has no curative treatment. Utilize the WHO mortality database and similar resources. |
| Standard of care | |
| Disease modification | Categorize the modification of "{disease}" {or disease synonyms} that the current standard of care provides into one of the following ordinal categories:  - 1 = curative/fully preventative  - 2 = high disease modification  - 3 = moderate disease modification  - 4 = low disease modification  - 5 = symptom relief only  Utilize sources such as PubMed and clinical guidelines to determine the current standard of care. |
| Adverse events | Categorize the level of side effects most commonly resulting from the administration of the current standard of care for "{disease}" {or disease synonyms} into one of the following ordinal categories:  - 1 = mild symptoms, no intervention required  - 2 = moderate symptoms, intervention required  - 3 = serious symptoms, intervention required  - 4 = very serious symptoms, intervention required  - 5 = serious adverse events often resulting in death  Deduct a point if the disease can be managed and/or resolved with lifestyle adjustments. |
| Route of administration | Categorize the route of administration of the current standard of care for "{disease}" {or disease synonyms} into one of the following ordinal categories:  - 1 = over the counter oral  - 2 = prescription oral  - 3 = injection/intravenous  - 4 = surgical procedure  - 5 = major surgical procedure |
| Frequency of administration | Categorize the frequency of administration of the current standard of care for "{disease}" {or disease synonyms} into one of the following ordinal categories:  - 1 = once only  - 2 = once per month (or less)  - 3 = once per week  - 4 = once per day  - 5 = multiple times per day  Degenerative diseases should score high. Utilize sources such as clinical guidelines and drug labels. |
| Accessibility | |
| Cost to patients | Categorize the cost to patients for the current standard of care for "{disease}" {or disease synonyms} into one of the following ordinal categories:  - 1 = very low ($10-100 per month, e.g. aspirin)  - 2 = low ($100-1000 per month e.g. generic prescription medicine)  - 3 = moderate ($1000-10,000 per month, e.g. on patent small molecule)  - 4 = high ($10,000-100,000 per month, e.g. established biologics)  - 5 = very high (>$100,000 per month, e.g. gene therapy) |
| Robust supply | Categorize the robustness of the supply chain for the standard of care for "{disease}" {or disease synonyms} into one of the following ordinal categories:  - 1 = very high (available worldwide, long shelf-life)  - 2 = high (available worldwide, occasional availability issues)  - 3 = moderate (only available in some countries OR needs cold chain)  - 4 = low (only available in some countries, reliability issues)  - 5 = very low (only available in few countries, single manufacturer, frequent stock-outs) |
| Regulatory barriers | Categorize the difficulty to acquire the standard of care for "{disease}" {or disease synonyms} posed by regulatory barriers into one of the following categories:  - 1 = Low (licensed to treat disease in at least 3 stringent regulatory regions)  - 3 = Moderate (licensed to treat the disease in only 1 stringent regulatory authority region)  - 5 = High (not licensed in any stringent authority region and only used off-label)  Utilize sources such as global clinical guidelines, FDA and other similar authorities. Disease treated with generic drugs should score low. |

**S5 Table. All eleven prompts used to generate unmet need scores across diseases. The prompt contents at the end of each prompt were added to correct for systematic biases in outputs during development.**
